# Determinants of accreditation perception among midwifery students: the role of knowledge and engagement

**DOI:** 10.1590/1980-220X-REEUSP-2026-0176en

**Published:** 2026-09-21

**Authors:** Serap Öner, Eylül Tutar

**Affiliations:** 1 Fırat University, Faculty of Health Sciences, Department of Midwifery, Elazığ, Türkiye.

**Keywords:** Midwifery, Accreditation, Total Quality Management, Students, Perception, Tocologia, Acreditação, Gestão da Qualidade Total, Estudantes, Percepção

## Abstract

**Objective:**

To examine midwifery students’ knowledge and perceptions of quality assurance and accreditation, and to identify factors associated with accreditation perception.

**Method:**

This cross-sectional study was conducted with 253 undergraduate midwifery students. Data were collected using a Personal Information Form, a Quality and Accreditation Knowledge Questionnaire, and the Accreditation Perception Scale. Descriptive statistics, t-tests, ANOVA, and multiple linear regression analysis were used.

**Results:**

Students demonstrated a moderate level of accreditation perception (mean = 3.42 ± 1.11). Knowledge of accreditation (β = 0.618, p < 0.001) and willingness to participate (β = 0.476, p = 0.002) were significant predictors, explaining 17.3% of the variance (R^2^ = 0.173). Year of study and participation in scientific activities were not significant (p > 0.05).

**Conclusion:**

Accreditation perception is mainly influenced by knowledge and engagement rather than academic progression. Integrating structured education on quality assurance and accreditation into curricula may enhance student awareness and support the development of a sustainable quality culture.

## RESUMO

**Objetivo:**

Examinar o conhecimento e as percepções de estudantes de assistência obstétrica sobre garantia da qualidade e acreditação e identificar fatores associados à percepção sobre acreditação.

**Método:**

Estudo transversal conduzido com 253 estudantes de graduação em assistência obstétrica. Os dados foram coletados por meio de um Formulário de Informações Pessoais, um Questionário de Conhecimento sobre Qualidade e Acreditação e a Escala de Percepção sobre Acreditação. Foram utilizadas estatísticas descritivas, testes t, ANOVA e análise de regressão linear múltipla.

**Resultados:**

Os estudantes apresentaram nível moderado de percepção sobre acreditação (média = 3,42 ± 1,11). O conhecimento sobre acreditação (β = 0,618; p < 0,001) e a disposição para participar (β = 0,476; p = 0,002) foram preditores significativos, explicando 17,3% da variância (R^2^ = 0,173). O ano de estudo e a participação em atividades científicas não foram preditores significativos (p > 0,05).

**Conclusão:**

A percepção sobre acreditação é influenciada principalmente pelo conhecimento e pelo engajamento, mais do que pela progressão acadêmica. A integração de educação estruturada sobre garantia da qualidade e acreditação aos currículos pode ampliar a conscientização dos estudantes e apoiar o desenvolvimento de uma cultura de qualidade sustentável.

## INTRODUCTION

Quality assurance and accreditation have become integral components of higher education systems worldwide, aiming to ensure transparency, accountability, and continuous improvement in educational quality. In health professions education, accreditation functions as a formal external review process that evaluates whether educational programmes meet predefined standards and adequately prepare graduates for safe, effective, and high-quality practice^(1)^. As healthcare systems increasingly emphasise patient safety and evidence-based care, the alignment of educational programmes with quality standards has gained critical importance.

International policy frameworks consistently highlight the central role of education quality in strengthening health systems. The WHO has underscored that robust accreditation mechanisms in health professions education contribute to workforce competence, service quality, and population health outcomes^(1,2)^. Similarly, global quality frameworks emphasise that accreditation promotes standardisation^(3)^, accountability, and a culture of continuous improvement by benchmarking educational programmes against national and international expectations. International frameworks emphasise that quality assurance systems are closely linked to educational outcomes and institutional performance^(4,5)^. Accreditation programmes have been associated with improved management practices and better organisational performance in healthcare settings, supporting their role in strengthening quality management systems^(6)^.

Within higher education institutions, students are increasingly recognised as key stakeholders in quality assurance and accreditation processes. Contemporary quality assurance models emphasise student engagement not only as a requirement for accountability but also as a driver of sustainable quality cultures in universities^(7,8)^. Evidence suggests that students with higher awareness of quality assurance processes demonstrate greater engagement in learning activities, provide more meaningful feedback, and contribute more actively to programme development and improvement^(9,10)^. Previous research published in nurse and midwifery education contexts has demonstrated that students’ engagement with institutional quality assurance processes is closely linked to their educational experiences and perceptions of programme quality^(11,12)^. Students are increasingly recognised as active partners in quality assurance processes rather than passive recipients of education^(13)^.

Quality in higher education is a multidimensional concept encompassing transformation, accountability, and continuous improvement^(14)^. In this context, accreditation should be understood not only as an institutional or regulatory process but also as a key component of a broader culture of quality and safety in healthcare education. This perspective emphasises continuous improvement, accountability, and active stakeholder engagement, including students as essential contributors to sustainable quality systems.

Health professions education literature further indicates that students’ perceptions of accreditation and quality initiatives influence their educational experiences and professional identity development. Studies conducted in nursing and allied health programmes have shown that students who understand accreditation standards report higher satisfaction with their education and greater confidence in programme outcomes^(15,16)^. These findings highlight the importance of assessing students’ awareness and perceptions as part of broader quality assurance efforts.

Midwifery education occupies a distinctive position within health professions education due to its direct and long-term impact on maternal and neonatal health outcomes. High-quality midwifery education has been associated with improved maternal and newborn outcomes, enhanced continuity of care, and reduced unnecessary interventions^(17)^. Accreditation of midwifery education programmes aims to ensure that graduates acquire the competencies, ethical values, and clinical skills required to deliver woman-centred, safe, and evidence-based care.

Despite the recognised importance of accreditation in health professions education, empirical evidence regarding midwifery students’ awareness of quality assurance and accreditation processes remains limited. Existing research has predominantly focused on programme outcomes, curriculum alignment, and faculty perspectives, while students’ knowledge, attitudes, and awareness have received comparatively less attention^(15)^. This gap is particularly evident in low- and middle-resource educational contexts, where institutional capacity and systematic student engagement in quality processes may be constrained.

In Turkey, higher education quality assurance is coordinated through a national framework aligned with European and international standards^(18)^. While accreditation initiatives have expanded across faculties of health sciences, the extent to which midwifery students are informed about, engaged in, and aware of quality and accreditation processes remains unclear. Given that students are central to the sustainability of quality cultures, understanding their awareness and perceptions is essential for strengthening accreditation practices and supporting continuous programme improvement.

Accreditation processes in healthcare settings have been shown to influence not only structural and procedural improvements but also professionals’ perceptions of quality, particularly in terms of outcomes and service effectiveness^(19)^, reinforcing the importance of accreditation in shaping quality perceptions.

Therefore, this study aimed to examine midwifery students’ knowledge of accreditation and their engagement in quality-related processes, and to identify determinants of accreditation perception. Despite the recognised importance of accreditation in health professions education, there remains a lack of empirical evidence regarding how students’ knowledge and engagement influence their perception of accreditation, particularly in midwifery education. In this context, the research question is: *What are the factors associated with midwifery students’ accreditation perception?* It was hypothesised that higher accreditation knowledge would be associated with more positive accreditation perceptions. Greater willingness to participate in accreditation processes was also expected to be positively associated with accreditation perception. In contrast, academic year and participation in scientific activities were not expected to be significant predictors.

## METHOD

### Study Design

This study used a descriptive cross-sectional design to examine midwifery students’ knowledge of accreditation and their engagement in quality-related processes. The study was reported in accordance with the Strengthening the Reporting of Observational Studies in Epidemiology (STROBE) guidelines for cross-sectional studies.

### Population and Sample

The study population consisted of undergraduate midwifery students enrolled in the first, second, third, and fourth academic years at a public university in eastern Turkey during the 2025–2026 academic year. No sampling method was applied, and all eligible students were invited to participate. A total of 253 students who completed the online questionnaire in full were included in the final analysis.

### Setting

The study was conducted at the Department of Midwifery of a public university in eastern Turkey.

### Selection Criteria

Students were eligible to participate if they were enrolled in the Department of Midwifery, were undergraduate students (1st–4th year), were able to understand the questionnaire, and agreed to participate voluntarily.

### Data Collection

Data were collected online between December 2025 and February 2026 after obtaining ethical approval and institutional permission. An online questionnaire was created using a secure web-based survey platform, and the survey link was distributed to eligible students through official institutional communication channels. Completion of the questionnaire took approximately 10–15 minutes. Participation was voluntary, and students were able to withdraw at any stage prior to submitting the questionnaire.

### Data Collection Instruments

Data were collected using a Personal Information Form, a Quality and Accreditation Knowledge Questionnaire, and the Accreditation Perception Scale. The Personal Information Form consisted of 10 items designed to collect sociodemographic information. The Quality and Accreditation Knowledge Questionnaire included 15 items assessing awareness of programme quality, accreditation status, and participation in quality-related activities. Students’ perceptions of accreditation were measured using the Accreditation Perception Scale developed by Semerci^(20)^, which consists of 17 items rated on a five-point Likert scale. In the present study, the scale’s internal consistency was excellent (Cronbach’s alpha = 0.98).

### Data Analysis and Treatment

Data were analysed using IBM SPSS Statistics version 22.0. Descriptive statistics (frequencies, percentages, means, and standard deviations) were used to summarise participants’ characteristics and scale scores. Associations between categorical variables were examined using the chi-square test. Independent-samples t-tests and one-way analysis of variance (ANOVA) were used to compare mean scores between groups. Multiple linear regression analysis was performed to identify factors associated with accreditation perception. Statistical significance was set at p < 0.05.

### Ethical Aspects

Ethical approval for the study was obtained from the Fırat University Non-Interventional Research Ethics Committee (date: 11 December 2025; decision no.: 2025/18-38). Institutional permission was also obtained from the Faculty of Health Sciences, Department of Midwifery. All participants were informed about the purpose of the study, and electronic informed consent was obtained prior to data collection. The study was conducted in accordance with the principles of the Declaration of Helsinki.

### Use of Artificial Intelligence

No artificial intelligence tools were used in the design, data collection, analysis, or interpretation of this study.

## RESULTS

### Participants’ Characteristics

A total of 253 undergraduate midwifery students participated in the study. The mean age of the participants was 20.52 ± 2.32 years. Regarding year of study, 28.7% of the students were first-year, 36.1% were second-year, 29.1% were third-year, and 6.1% were fourth-year students. In terms of knowledge about accreditation, 50.2% of the students reported that they knew the meaning of accreditation, while 49.8% reported not knowing its meaning. Participants’ characteristics are presented in Table 1.

**Table 1:** Participants’ characteristics (n = 253) – Elazığ, Turkey, 2025-2026.

Variables	n	%
Year of study		
1st year	73	28.7
2nd year	91	36.1
3rd year	74	29.1
4th year	15	6.1
Knowledge of the meaning of accreditation		
Yes	127	50.2
No	126	49.8

### Reliability of the Scale

The Accreditation Perception Scale demonstrated excellent internal consistency in the present study. The Cronbach’s alpha coefficient was 0.98 for the total scale, 0.96 for the Quality Assurance subscale, and 0.97 for the Quality Evaluation subscale.

### Accreditation Perception Scores

The mean total score of the Accreditation Perception Scale was 3.42 ± 1.11, indicating a moderate level of accreditation perception among midwifery students. Regarding the subscales, the mean score for the Quality Assurance dimension was 3.38 ± 1.14, while the mean score for the Quality Evaluation dimension was 3.45 ± 1.15. Accreditation perception scores are presented in Table 2. The Quality Evaluation subscale yielded slightly higher scores compared to the Quality Assurance subscale.

**Table 2:** Mean scores of the Accreditation Perception Scale and subscales (n = 253) – Elazığ, Turkey, 2025-2026.

Scale/Subscale	Mean ± SD
Total Accreditation Perception	3.42 ± 1.11
Quality Assurance	3.38 ± 1.14
Quality Evaluation	3.45 ± 1.15

As presented in Table 3, students demonstrated moderate awareness of accreditation processes but a relatively high willingness to participate. While knowledge of accreditation was limited, most students expressed positive attitudes toward accreditation and its perceived benefits. Differences in total Accreditation Perception Scale scores were examined according to students’ year of study using one-way analysis of variance (ANOVA). The analysis revealed no statistically significant difference in accreditation perception scores across year levels (F = 2.45, p = 0.065).

**Table 3:** Midwifery students’ knowledge, attitudes, and awareness regarding accreditation (n = 253) – Elazığ, Turkey, 2025-2026.

Variables	Categories	n	%
Knowledge of accreditation	Yes	127	50.2
	No	126	49.8
Awareness of departmental accreditation activities	Yes	62	24.5
	No	191	75.5
Willingness to participate in accreditation activities	Yes	172	68.0
	No	81	32.0
Perceived benefits of accreditation for students and graduates	Yes	134	53.0
	No	1	0.4
	Undecided	118	46.6
Participation in scientific activities	Yes	81	32.0
	No	172	68.0

Total accreditation perception scores were compared between students who reported participating in scientific activities and those who reported no participation using an independent-samples t-test. The difference between the two groups was not statistically significant (t = 1.44, p = 0.154). A statistically significant difference was found between students who reported knowing the meaning of accreditation and those who did not. Students who were knowledgeable about accreditation demonstrated significantly higher accreditation perception scores compared to those who were not (t = 5.06, p < 0.001). These comparisons are presented in Table 4.

**Table 4:** Comparison of Accreditation Perception Scale scores according to selected variables (n = 253) – Elazığ, Turkey, 2025-2026.

Variables	Test	Statistic	p-value
Year of study	ANOVA	F = 2.45	0.065
Participation in scientific activities	t-test	t = 1.44	0.154
Knowledge of the meaning of accreditation	t-test	t = 5.06	< 0.001

### Factors Associated with Accreditation Perception

A multiple linear regression analysis was conducted to identify factors associated with midwifery students’ perceptions of accreditation. The overall model was statistically significant (F = 9.01, p < 0.001) and explained 17.3% of the variance in accreditation perception (R^2^ = 0.173). Knowledge of accreditation was a significant predictor of accreditation perception (β = 0.618, p < 0.001). Willingness to participate in accreditation processes was also a significant predictor (β = 0.476, p = 0.002). Awareness of departmental accreditation activities showed a marginal association (β = 0.336, p = 0.073). Year of study (p = 0.462) and participation in scientific activities (p = 0.749) were not significantly associated with accreditation perception (Table 5). The regression model indicates that knowledge and willingness to participate are the most important predictors of accreditation perception.

**Table 5:** Factors associated with accreditation perception (multiple linear regression analysis) – Elazığ, Turkey, 2025-2026.

Variables	β	p-value	95% CI
Knowledge of accreditation	0.618	<0.001	0.35–0.89
Willingness to participate in accreditation processes	0.476	0.002	0.18–0.78
Awareness of departmental accreditation activities	0.336	0.073	−0.03–0.70
Year of study	–	0.462	–
Participation in scientific activities	–	0.749	–

Model statistics: R^2^ = 0.173; F = 9.01; p < 0.001.

## DISCUSSION

The findings of this study highlight a noteworthy discrepancy between midwifery students’ knowledge of accreditation and their awareness of departmental accreditation activities. As shown in Table 3, approximately half of the students reported being familiar with the concept of accreditation, yet only one-quarter were aware of ongoing accreditation processes within their own department. This gap suggests that accreditation may be perceived as an abstract or institutional-level concept rather than a student-engaged, practice-oriented process. Similar observations have been reported in nursing and health professions education, where students demonstrate limited awareness of accreditation activities despite studying within accredited or accreditation-seeking programmes^(15,16)^. This aligns with broader higher education perspectives that link quality assurance to improved institutional performance and educational outcomes^(4)^. These findings further support the notion that accreditation is not merely a formal evaluation process but an integral element of a broader quality and safety culture within health professions education.

Despite limited awareness, students in the present study expressed a strong willingness to participate in accreditation-related activities, with more than two-thirds indicating readiness to engage (Table 3). This finding is particularly important, as contemporary quality assurance frameworks emphasise student participation as a cornerstone of sustainable quality cultures in higher education^(7,8)^. The coexistence of low awareness and high willingness suggests an underutilised potential for student involvement in quality assurance processes. Structured opportunities for participation, such as student representation in quality committees, targeted training sessions, and integration of accreditation topics into the curriculum, may help bridge this gap. This finding is consistent with previous studies published in Nurse Education in Practice, which emphasise that students often hold positive attitudes toward quality-related initiatives even when their direct involvement in formal quality assurance processes is limited^(11,12)^.

Another notable finding concerns students’ participation in scientific activities, which was relatively low in the present sample (Table 3). Limited engagement in scientific and academic activities may restrict students’ exposure to broader quality improvement and evidence-based practice frameworks, including accreditation. Previous research has demonstrated that participation in academic and scholarly activities enhances students’ understanding of quality standards and strengthens professional identity development in nursing and midwifery education^(9,10,21)^. Therefore, increasing opportunities for student involvement in scientific events, workshops, and quality-focused projects may indirectly contribute to improved accreditation awareness.

Furthermore, more than half of the students perceived accreditation as beneficial for both students and graduates (Table 3), indicating generally positive attitudes toward quality assurance processes. Positive perceptions of accreditation have been associated with higher educational satisfaction and greater confidence in programme outcomes in health professions education^(15)^. However, the substantial proportion of undecided students suggests a need for clearer communication regarding the tangible outcomes and practical implications of accreditation for students’ educational experiences and future professional practice.

Taken together, these findings underscore the importance of embedding accreditation and quality assurance concepts more explicitly within midwifery education. Enhancing students’ awareness and active engagement in accreditation processes may not only strengthen quality cultures within institutions but also contribute to the preparation of midwives who are more attuned to quality improvement, accountability, and evidence-based practice in healthcare settings. These findings also highlight the importance of active learning approaches and structured student engagement in fostering a sustainable quality culture within health professions education.

In addition to students’ descriptive knowledge and awareness, the findings related to accreditation perception provide further insight into how midwifery students conceptualise quality assurance in education. The results of the Accreditation Perception Scale indicated a generally moderate to positive perception of accreditation among students. This suggests that, even in the absence of comprehensive formal information, students tend to associate accreditation with educational quality, accountability, and improvement. Similar patterns have been reported in previous studies conducted with nursing and health sciences students, in which accreditation was perceived positively despite limited direct engagement in accreditation processes^(15,20)^.

The relatively positive perception of accreditation observed in this study may reflect students’ broader expectations regarding educational quality and professional competence. Accreditation is often viewed by students as an indicator of institutional credibility and graduate value in the labour market, which may explain favourable attitudes even when procedural knowledge is limited. However, the lack of detailed awareness highlighted in Table 3 suggests that these perceptions may be based more on general beliefs than on concrete understanding. This disconnect underscores the importance of integrating accreditation-related content into formal coursework and experiential learning opportunities to ensure that students’ perceptions are informed and aligned with practice. It should be noted that perception does not necessarily reflect actual competence or performance, and therefore, the findings should be interpreted within a perceptual and educational framework.

The findings of the regression analysis provide a deeper understanding of the determinants of accreditation perception among midwifery students. Knowledge of accreditation emerged as the strongest predictor, reinforcing the central role of awareness in shaping students’ perceptions of quality assurance processes. This finding is consistent with the notion that understanding quality processes is fundamental to meaningful engagement in higher education^(14)^. The regression coefficients indicate that increases in students’ knowledge of accreditation and their willingness to participate are associated with higher levels of accreditation perception. However, the model demonstrated a moderate explanatory power (R^2^ = 0.173), suggesting that other factors may also contribute to accreditation perception. Future research may explore additional variables such as clinical exposure, institutional context, and experiential learning opportunities to provide a more comprehensive understanding of the determinants of accreditation perception.

This finding indicates that accreditation perception is largely knowledge-driven rather than experience-driven, as neither academic progression nor participation in scientific activities significantly influenced perception levels. This supports the notion that accreditation-related competencies do not develop passively over time but require structured educational interventions.

Furthermore, students’ willingness to participate in accreditation processes was identified as a significant predictor, highlighting the importance of student engagement in fostering positive attitudes toward quality assurance. This finding aligns with contemporary quality frameworks that position students as active stakeholders in institutional quality processes.

This study has several strengths, including a relatively large sample size and the use of a validated measurement tool with excellent internal consistency. However, some limitations should be acknowledged. The cross-sectional design precludes causal inferences, and the single-centre setting may limit the generalisability of the findings. In addition, data were based on self-reported measures, which may be subject to response bias. In addition, the regression model demonstrated moderate explanatory power, indicating that other unmeasured variables may also influence accreditation perception.

From a practical perspective, the findings highlight the importance of integrating structured education on quality assurance and accreditation into undergraduate midwifery curricula. Enhancing students’ knowledge and actively engaging them in accreditation processes may contribute to the development of a sustainable quality culture within educational institutions. Creating opportunities for student participation in quality-related activities, such as programme evaluation and feedback systems, may further strengthen both learning experiences and institutional quality improvement efforts. These findings indicate that accreditation literacy can be effectively taught as part of undergraduate education. Furthermore, student engagement in quality assurance processes can be systematically structured and supported, and active participation in such processes may contribute to students’ professional development.

Accreditation-related knowledge may be integrated into curricula through structured courses or modules, while student engagement can be promoted through participation in quality committees and experiential learning opportunities.

This study contributes to the existing literature by highlighting the role of knowledge and student engagement as key determinants of accreditation perception in midwifery education, an area that has received limited empirical attention. Unlike previous studies that primarily focused on institutional or programme-level outcomes, this study provides a student-centred perspective on accreditation processes. The findings offer practical implications for integrating accreditation-related content into curricula and promoting active student participation in quality assurance processes, thereby supporting the development of a sustainable quality culture in health professions education.

These findings emphasise the need for structured educational strategies that enhance students’ knowledge of accreditation and actively involve them in quality assurance processes. Strengthening student engagement in accreditation may not only improve educational experiences but also foster a sustainable quality culture and better health outcomes.

## CONCLUSION

This study demonstrated that midwifery students have a moderate level of accreditation perception. Knowledge of accreditation and willingness to participate in accreditation processes were identified as significant predictors of accreditation perception, whereas academic year and participation in scientific activities were not associated with perception levels. These findings indicate that accreditation perception is primarily shaped by knowledge and engagement rather than academic progression. Strengthening students’ knowledge and engagement may contribute to the development of a more quality-oriented educational environment in midwifery education.

## Data Availability

The data supporting the findings of this study are available from the corresponding author upon reasonable request.
