# Supplementary material for: Determinants of accreditation perception among midwifery students: the role of knowledge and engagement
Source: Rev Esc Enferm USP. 2026 Sep 21;60:e20260176. doi: 10.1590/1980-220X-REEUSP-2026-0176en (PMC13597090; doi:10.1590/1980-220X-REEUSP-2026-0176en)
Supplement: Translation

# Translation: Factores determinantes de la percepción de la acreditación entre las estudiantes de partería: el papel del conocimiento y el compromiso

**DOI:** 10.1590/1980-220X-REEUSP-2026-0176es

**Authors:** Serap Öner, Eylül Tutar

**Affiliations:** 1 Fırat University, Faculty of Health Sciences, Department of Midwifery, Elazığ, Türkiye.

**Keywords:** Partería, Acreditación, Gestión de la Calidad Total, Estudiantes, Percepción

## Abstract

**Objetivo:**

Examinar los conocimientos y las percepciones de las estudiantes de partería sobre el aseguramiento de la calidad y la acreditación, e identificar los factores asociados con la percepción de la acreditación.

**Método:**

Este estudio transversal se llevó a cabo con 253 estudiantes de pregrado de partería. Los datos se recolectaron mediante un formulario de información personal, un cuestionario de conocimientos sobre calidad y acreditación, y la Escala de Percepción de la Acreditación. Se utilizaron estadísticas descriptivas, pruebas t, ANOVA y análisis de regresión lineal múltiple.

**Resultados:**

Las estudiantes mostraron un nivel moderado de percepción de la acreditación (media = 3,42 ± 1,11). El conocimiento sobre la acreditación (β = 0,618, p < 0,001) y la disposición a participar (β = 0,476, p = 0,002) fueron predictores significativos, que explicaron el 17,3 % de la varianza (R^2^ = 0,173). El año de estudio y la participación en actividades científicas no resultaron significativos (p > 0,05).

**Conclusión:**

La percepción de la acreditación se ve influida principalmente por el conocimiento y el compromiso, más que por el avance académico. La integración de una formación estructurada sobre garantía de calidad y acreditación en los planes de estudio podría aumentar la concientización de los estudiantes y apoyar el desarrollo de una cultura de calidad sostenible.

## INTRODUCCIÓN

El aseguramiento de la calidad y la acreditación se han convertido en componentes integrales de los sistemas de educación superior en todo el mundo, con el objetivo de garantizar la transparencia, la rendición de cuentas y la mejora continua de la calidad educativa. En la educación de las profesiones de la salud, la acreditación funciona como un proceso formal de revisión externa que evalúa si los programas educativos cumplen con estándares predefinidos y preparan adecuadamente a los graduados para una práctica segura, eficaz y de alta calidad^(1)^. A medida que los sistemas de atención de salud hacen cada vez más hincapié en la seguridad del paciente y la atención basada en la evidencia, la alineación de los programas educativos con los estándares de calidad ha adquirido una importancia fundamental.

Los marcos de políticas internacionales destacan sistemáticamente el papel central de la calidad de la educación en el fortalecimiento de los sistemas de salud. La OMS ha subrayado que los mecanismos sólidos de acreditación en la educación de las profesiones de la salud contribuyen a la competencia del personal, la calidad del servicio y los resultados de salud de la población^(1,2)^. Del mismo modo, los marcos globales de calidad enfatizan que la acreditación promueve la estandarización^(3)^, la rendición de cuentas y una cultura de mejora continua al comparar los programas educativos con las expectativas nacionales e internacionales. Los marcos internacionales hacen hincapié en que los sistemas de garantía de calidad están estrechamente vinculados a los resultados educativos y al desempeño institucional^(4,5)^. Los programas de acreditación se han asociado con mejores prácticas de gestión y un mejor desempeño organizacional en los entornos de atención de la salud, lo que respalda su papel en el fortalecimiento de los sistemas de gestión de la calidad^(6)^.

Dentro de las instituciones de educación superior, se reconoce cada vez más a los estudiantes como partes interesadas clave en los procesos de garantía de calidad y acreditación. Los modelos contemporáneos de garantía de calidad hacen hincapié en la participación de los estudiantes no solo como un requisito para la rendición de cuentas, sino también como un motor de culturas de calidad sostenibles en las universidades^(7,8)^. La evidencia sugiere que los estudiantes con mayor conciencia de los procesos de garantía de calidad demuestran una mayor participación en las actividades de aprendizaje, brindan retroalimentación más significativa y contribuyen más activamente al desarrollo y la mejora de los programas^(9,10)^. Investigaciones previas publicadas en el contexto de la formación de enfermería y partería han demostrado que la participación de los estudiantes en los procesos institucionales de garantía de calidad está estrechamente vinculada a sus experiencias educativas y a sus percepciones sobre la calidad de los programas^(11,12)^. Cada vez más se reconoce a los estudiantes como socios activos en los procesos de garantía de calidad, en lugar de como receptores pasivos de la educación^(13)^.

La calidad en la educación superior es un concepto multidimensional que abarca la transformación, la rendición de cuentas y la mejora continua^(14)^. En este contexto, la acreditación debe entenderse no solo como un proceso institucional o regulatorio, sino también como un componente clave de una cultura más amplia de calidad y seguridad en la educación en el ámbito de la salud. Esta perspectiva hace hincapié en la mejora continua, la rendición de cuentas y la participación activa de las partes interesadas, incluyendo a los estudiantes como contribuyentes esenciales a los sistemas de calidad sostenibles.

La literatura sobre la educación en profesiones de la salud indica además que las percepciones de los estudiantes respecto a la acreditación y las iniciativas de calidad influyen en sus experiencias educativas y en el desarrollo de su identidad profesional. Estudios realizados en programas de enfermería y profesiones afines han demostrado que los estudiantes que comprenden los estándares de acreditación reportan una mayor satisfacción con su educación y una mayor confianza en los resultados del programa^(15,16)^. Estos hallazgos resaltan la importancia de evaluar la conciencia y las percepciones de los estudiantes como parte de esfuerzos más amplios de garantía de calidad.

La formación en partería ocupa una posición distintiva dentro de la educación de las profesiones de la salud debido a su impacto directo y a largo plazo en los resultados de salud materna y neonatal. La formación en partería de alta calidad se ha asociado con mejores resultados maternos y neonatales, una mayor continuidad de la atención y una reducción de las intervenciones innecesarias^(17)^. La acreditación de los programas de formación en partería tiene como objetivo garantizar que las graduadas adquieran las competencias, los valores éticos y las habilidades clínicas necesarias para brindar una atención centrada en la mujer, segura y basada en la evidencia.

A pesar de la reconocida importancia de la acreditación en la formación de las profesiones de la salud, la evidencia empírica sobre la conciencia de las estudiantes de partería respecto a los procesos de garantía de calidad y acreditación sigue siendo limitada. Las investigaciones existentes se han centrado predominantemente en los resultados de los programas, la alineación curricular y las perspectivas del cuerpo docente, mientras que los conocimientos, las actitudes y la conciencia de las estudiantes han recibido comparativamente menos atención^(15)^. Esta brecha es particularmente evidente en contextos educativos de recursos bajos y medios, donde la capacidad institucional y la participación sistemática de las estudiantes en los procesos de calidad pueden verse limitadas.

En Turquía, el aseguramiento de la calidad de la educación superior se coordina a través de un marco nacional alineado con las normas europeas e internacionales^(18)^. Si bien las iniciativas de acreditación se han extendido por las facultades de ciencias de la salud, aún no está claro en qué medida los estudiantes de partería están informados, participan y son conscientes de los procesos de calidad y acreditación. Dado que los estudiantes son fundamentales para la sostenibilidad de las culturas de calidad, comprender su conciencia y sus percepciones es esencial para fortalecer las prácticas de acreditación y apoyar la mejora continua de los programas.

Se ha demostrado que los procesos de acreditación en entornos de atención de la salud influyen no solo en las mejoras estructurales y de procedimientos, sino también en las percepciones de los profesionales sobre la calidad, particularmente en términos de resultados y eficacia del servicio^(19)^, lo que refuerza la importancia de la acreditación en la configuración de las percepciones de calidad.

Por lo tanto, este estudio tuvo como objetivo examinar el conocimiento que tienen las estudiantes de partería sobre la acreditación y su participación en los procesos relacionados con la calidad, así como identificar los factores determinantes de su percepción de la acreditación. A pesar de la reconocida importancia de la acreditación en la formación de las profesiones de la salud, sigue habiendo una falta de evidencia empírica sobre cómo los conocimientos y la participación de los estudiantes influyen en su percepción de la acreditación, particularmente en la formación en partería. En este contexto, la pregunta de investigación es: *¿Cuáles son los factores asociados con la percepción de la acreditación por parte de los estudiantes de partería?* Se planteó la hipótesis de que un mayor conocimiento sobre la acreditación se asociaría con percepciones más positivas de la misma. También se esperaba que una mayor disposición a participar en los procesos de acreditación se asociara positivamente con la percepción de la acreditación. Por el contrario, no se esperaba que el año académico ni la participación en actividades científicas fueran predictores significativos.

## MÉTODO

### Diseño del Estudio

Este estudio utilizó un diseño descriptivo transversal para examinar el conocimiento que tienen las estudiantes de partería sobre la acreditación y su participación en procesos relacionados con la calidad. El estudio se presentó de acuerdo con las directrices STROBE (Fortalecimiento de la presentación de informes de estudios observacionales en epidemiología) para estudios transversales.

### Población y Muestra

La población del estudio estuvo compuesta por estudiantes de pregrado de partería matriculados en el primer, segundo, tercer y cuarto año académico en una universidad pública del este de Turquía durante el año académico 2025–2026. No se aplicó ningún método de muestreo y se invitó a participar a todos los estudiantes elegibles. Se incluyeron en el análisis final un total de 253 estudiantes que completaron el cuestionario en línea en su totalidad.

### Entorno

El estudio se llevó a cabo en el Departamento de Partería de una universidad pública del este de Turquía.

### Criterios de Selección

Las estudiantes eran elegibles para participar si estaban inscritas en el Departamento de Partería, eran estudiantes de pregrado (1.º a 4.º año), podían comprender el cuestionario y aceptaban participar voluntariamente.

### Recolección de Datos

Los datos se recolectaron en línea entre diciembre de 2025 y febrero de 2026, tras obtener la aprobación ética y el permiso institucional. Se creó un cuestionario en línea utilizando una plataforma segura de encuestas basada en la web, y el enlace a la encuesta se distribuyó a los estudiantes elegibles a través de los canales oficiales de comunicación de la institución. Completar el cuestionario tomaba aproximadamente entre 10 y 15 minutos. La participación era voluntaria, y los estudiantes podían retirarse en cualquier momento antes de enviar el cuestionario.

### Instrumentos de Recolección de Datos

Los datos se recolectaron mediante un Formulario de información personal, un Cuestionario de conocimientos sobre calidad y acreditación y la Escala de percepción de la acreditación. El Formulario de información personal constaba de 10 ítems diseñados para recolectar información sociodemográfica. El Cuestionario de conocimientos sobre calidad y acreditación (Quality and Accreditation Knowledge Questionnaire) incluía 15 ítems que evaluaban el conocimiento sobre la calidad del programa, el estado de acreditación y la participación en actividades relacionadas con la calidad. Las percepciones de los estudiantes sobre la acreditación se midieron mediante la Escala de Percepción de la Acreditación (Accreditation Perception Scale) desarrollada por Semerci^(20)^, que consta de 17 ítems calificados en una escala de Likert de cinco puntos. En el presente estudio, la consistencia interna de la escala fue excelente (alfa de Cronbach = 0,98).

### Análisis y Tratamiento de Datos

Los datos se analizaron con IBM SPSS Statistics versión 22.0. Se utilizaron estadísticas descriptivas (frecuencias, porcentajes, promedios y desviaciones estándar) para resumir las características de los participantes y los puntajes de las escalas. Las asociaciones entre variables categóricas se examinaron mediante la prueba de chi-cuadrado. Se utilizaron pruebas t para muestras independientes y análisis de varianza unidireccional (ANOVA) para comparar los promedios de los puntajes entre los grupos. Se realizó un análisis de regresión lineal múltiple para identificar los factores asociados con la percepción de la acreditación. La significación estadística se fijó en p < 0,05.

### Aspectos Éticos

La aprobación ética del estudio fue otorgada por el Comité de Ética de Investigación No Intervencionista de la Universidad de Fırat (fecha: 11 de diciembre de 2025; número de decisión: 2025/18-38). También se obtuvo el permiso institucional de la Facultad de Ciencias de la Salud, Departamento de Partería. Se informó a todas las participantes sobre el propósito del estudio y se obtuvo el consentimiento informado electrónico antes de la recolección de datos. El estudio se llevó a cabo de conformidad con los principios de la Declaración de Helsinki.

### Uso de Inteligencia Artificial

No se utilizaron herramientas de inteligencia artificial en el diseño, la recolección de datos, el análisis ni la interpretación de este estudio.

## RESULTADOS

### Características de los Participantes

Participaron en el estudio un total de 253 estudiantes de pregrado de partería. La edad promedio de los participantes fue de 20,52 ± 2,32 años. En cuanto al año de estudio, el 28,7 % de los estudiantes cursaba el primer año, el 36,1 % el segundo, el 29,1 % el tercero y el 6,1 % el cuarto. En cuanto al conocimiento sobre la acreditación, el 50,2 % de las estudiantes indicaron que conocían el significado de la acreditación, mientras que el 49,8 % indicaron que no lo conocían. Las características de las participantes se presentan en la Tabla 1.

**Tabla 1:** Características de los participantes (n = 253) – Elazığ, Turquía, 2025-2026.

Variables	n	%
Curso de estudios		
1.º curso	73	28,7
2.º curso	91	36,1
3.º curso	74	29,1
4.º curso	15	6,1
Conocimiento del significado de la acreditación		
Sí	127	50,2
No	126	49,8

### Fiabilidad de la Escala

La Escala de Percepción de la Acreditación demostró una excelente consistencia interna en el presente estudio. El coeficiente alfa de Cronbach fue de 0,98 para la escala total, de 0,96 para la subescala de Garantía de Calidad y de 0,97 para la subescala de Evaluación de la Calidad.

### Puntuaciones de Percepción de la Acreditación

La puntuación media total de la Escala de Percepción de la Acreditación fue de 3,42 ± 1,11, lo que indica un nivel moderado de percepción de la acreditación entre las estudiantes de obstetricia. En cuanto a las subescalas, la puntuación media de la dimensión «Garantía de calidad» fue de 3,38 ± 1,14, mientras que la de la dimensión «Evaluación de la calidad» fue de 3,45 ± 1,15. Las puntuaciones de percepción de la acreditación se presentan en la Tabla 2. La subescala «Evaluación de la calidad» obtuvo puntuaciones ligeramente superiores en comparación con la subescala «Garantía de calidad».

**Tabla 2:** Puntuaciones medias de la Escala de Percepción de la Acreditación y sus subescalas (n = 253), Elazığ – Turquía, 2025-2026.

Escala/Subescala	Media ± DE
Percepción global de la acreditación	3,42 ± 1,11
Garantía de calidad	3,38 ± 1,14
Evaluación de la calidad	3,45 ± 1,15

Tal y como se muestra en la Tabla 3, los estudiantes demostraron un conocimiento moderado de los procesos de acreditación, pero una disposición relativamente alta a participar. Aunque el conocimiento sobre la acreditación era limitado, la mayoría de los estudiantes expresaron actitudes positivas hacia la acreditación y los beneficios que percibían de la misma. Se examinaron las diferencias en las puntuaciones totales de la Escala de Percepción de la Acreditación según el curso académico de los estudiantes mediante un análisis de varianza unidireccional (ANOVA). El análisis no reveló ninguna diferencia estadísticamente significativa en las puntuaciones de percepción de la acreditación entre los distintos cursos (F = 2,45; p = 0,065).

**Tabla 3:** Conocimientos, actitudes y concienciación de los estudiantes de obstetricia respecto a la acreditación (n = 253) – Elazığ, Turquía, 2025-2026.

Variables	Categorías	n	%
Conocimientos sobre la acreditación	Sí	127	50,2
	No	126	49,8
Conocimiento de las actividades de acreditación del departamento	Sí	62	24,5
	No	191	75,5
Disposición a participar en actividades de acreditación	Sí	172	68,0
	No	81	32,0
Beneficios percibidos de la acreditación para los estudiantes y titulados	Sí	134	53,0
	No	1	0,4
	Indeciso	118	46,6
Participación en actividades científicas	Sí	81	32,0
	No	172	68,0

Se compararon las puntuaciones totales de percepción de la acreditación entre los estudiantes que declararon participar en actividades científicas y aquellos que declararon no participar, utilizando una prueba t para muestras independientes. La diferencia entre los dos grupos no fue estadísticamente significativa (t = 1,44; p = 0,154). Se observó una diferencia estadísticamente significativa entre los estudiantes que declararon conocer el significado de la acreditación y aquellos que no lo conocían. Los estudiantes que conocían el concepto de acreditación obtuvieron puntuaciones significativamente más altas en la percepción de la acreditación en comparación con los que no lo conocían (t = 5,06; p < 0,001). Estas comparaciones se presentan en la Tabla 4.

**Tabla 4:** Comparación de las puntuaciones de la Escala de Percepción de la Acreditación según variables seleccionadas (n = 253) – Elazığ, Turquía, 2025-2026.

Variables	Prueba	Estadística	Valor p
Año de estudio	ANOVA	F = 2,45	0,065
Participación en actividades científicas	Prueba t	t = 1,44	0,154
Conocimiento del significado de la acreditación	prueba t	t = 5,06	< 0,001

### Factores Asociados a la Percepción de la Acreditación

Se llevó a cabo un análisis de regresión lineal múltiple para identificar los factores asociados a la percepción que tienen las estudiantes de obstetricia sobre la acreditación. El modelo global fue estadísticamente significativo (F = 9,01; p < 0,001) y explicó el 17,3 % de la varianza en la percepción de la acreditación (R^2^ = 0,173). El conocimiento sobre la acreditación fue un predictor significativo de la percepción de la acreditación (β = 0,618, p < 0,001). La disposición a participar en los procesos de acreditación también fue un predictor significativo (β = 0,476, p = 0,002). El conocimiento de las actividades de acreditación del departamento mostró una asociación marginal (β = 0,336, p = 0,073). El curso de estudios (p = 0,462) y la participación en actividades científicas (p = 0,749) no se asociaron de forma significativa con la percepción de la acreditación (Tabla 5). El modelo de regresión indica que el conocimiento y la disposición a participar son los predictores más importantes de la percepción de la acreditación.

**Tabla 5:** Factores asociados a la percepción de la acreditación (análisis de regresión lineal múltiple) – Elazığ, Turquía, 2025-2026.

Variables	β	Valor p	IC del 95 %
Conocimiento de la acreditación	0,618	<0,001	0,35–0,89
Disposición a participar en procesos de acreditación	0,476	0,002	0,18–0,78
Conocimiento de las actividades de acreditación del departamento	0,336	0,073	−0,03–0,70
Curso académico	–	0,462	–
Participación en actividades científicas	–	0,749	–

Estadísticas del modelo: R^2^ = 0,173; F = 9,01; p < 0,001.

## DISCUSIÓN

Los resultados de este estudio ponen de relieve una discrepancia notable entre el conocimiento que tienen las estudiantes de partería sobre la acreditación y su conocimiento de las actividades de acreditación del departamento. Como se muestra en la Tabla 3, aproximadamente la mitad de las estudiantes indicaron estar familiarizadas con el concepto de acreditación; sin embargo, solo una cuarta parte estaba al tanto de los procesos de acreditación en curso dentro de su propio departamento. Esta brecha sugiere que la acreditación podría percibirse como un concepto abstracto o de nivel institucional, en lugar de un proceso en el que participan los estudiantes y que está orientado a la práctica. Se han reportado observaciones similares en la formación de enfermería y otras profesiones de la salud, donde los estudiantes demuestran un conocimiento limitado de las actividades de acreditación a pesar de estudiar en programas acreditados o que buscan la acreditación^(15,16)^. Esto concuerda con perspectivas más amplias de la educación superior que vinculan la garantía de calidad con un mejor desempeño institucional y mejores resultados educativos^(4)^. Estos hallazgos respaldan aún más la idea de que la acreditación no es meramente un proceso de evaluación formal, sino un elemento integral de una cultura más amplia de calidad y seguridad dentro de la formación en profesiones de la salud.

A pesar del escaso conocimiento, los estudiantes del presente estudio expresaron una fuerte disposición a participar en actividades relacionadas con la acreditación, y más de dos tercios indicaron estar listos para participar (Tabla 3). Este hallazgo es particularmente importante, ya que los marcos contemporáneos de garantía de calidad enfatizan la participación estudiantil como piedra angular de las culturas de calidad sostenibles en la educación superior^(7,8)^. La coexistencia de un bajo nivel de concientización y una alta disposición sugiere un potencial subutilizado para la participación de los estudiantes en los procesos de garantía de calidad. Las oportunidades estructuradas de participación, tales como la representación estudiantil en comités de calidad, sesiones de capacitación específicas y la integración de temas de acreditación en el plan de estudios, pueden ayudar a cerrar esta brecha. Este hallazgo concuerda con estudios previos publicados en *Nurse Education in Practice*, que enfatizan que los estudiantes suelen tener actitudes positivas hacia las iniciativas relacionadas con la calidad, incluso cuando su participación directa en los procesos formales de garantía de calidad es limitada^(11,12)^.

Otro hallazgo notable se refiere a la participación de los estudiantes en actividades científicas, la cual fue relativamente baja en la presente muestra (Tabla 3). La participación limitada en actividades científicas y académicas puede restringir la exposición de los estudiantes a marcos más amplios de mejora de la calidad y de práctica basada en la evidencia, incluida la acreditación. Investigaciones previas han demostrado que la participación en actividades académicas y de investigación mejora la comprensión de los estudiantes sobre los estándares de calidad y fortalece el desarrollo de la identidad profesional en la educación en enfermería y partería^(9,10,21)^. Por lo tanto, aumentar las oportunidades para que los estudiantes participen en eventos científicos, talleres y proyectos centrados en la calidad podría contribuir indirectamente a una mayor concientización sobre la acreditación.

Además, más de la mitad de los estudiantes percibían la acreditación como beneficiosa tanto para los estudiantes como para los graduados (Tabla 3), lo que indica actitudes generalmente positivas hacia los procesos de garantía de calidad. Las percepciones positivas de la acreditación se han asociado con una mayor satisfacción educativa y una mayor confianza en los resultados de los programas en la formación de profesiones de la salud^(15)^. Sin embargo, la proporción considerable de estudiantes indecisos sugiere la necesidad de una comunicación más clara sobre los resultados tangibles y las implicaciones prácticas de la acreditación para las experiencias educativas de los estudiantes y su futura práctica profesional.

En conjunto, estos hallazgos subrayan la importancia de integrar de manera más explícita los conceptos de acreditación y garantía de calidad en la formación en partería. Mejorar la concientización y la participación activa de los estudiantes en los procesos de acreditación no solo podría fortalecer las culturas de calidad dentro de las instituciones, sino también contribuir a la preparación de parteras más sensibilizadas con la mejora de la calidad, la rendición de cuentas y la práctica basada en la evidencia en los entornos de atención de salud. Estos hallazgos también resaltan la importancia de los enfoques de aprendizaje activo y la participación estructurada de los estudiantes para fomentar una cultura de calidad sostenible en la educación de las profesiones de la salud.

Además del conocimiento descriptivo y la conciencia de los estudiantes, los hallazgos relacionados con la percepción de la acreditación brindan una perspectiva adicional sobre cómo los estudiantes de partería conceptualizan el aseguramiento de la calidad en la educación. Los resultados de la Escala de Percepción de la Acreditación indicaron una percepción generalmente moderada a positiva de la acreditación entre los estudiantes. Esto sugiere que, incluso ante la falta de información formal exhaustiva, los estudiantes tienden a asociar la acreditación con la calidad educativa, la rendición de cuentas y la mejora. Se han reportado patrones similares en estudios previos realizados con estudiantes de enfermería y ciencias de la salud, en los que la acreditación se percibía de manera positiva a pesar de la participación directa limitada en los procesos de acreditación^(15,20)^.

La percepción relativamente positiva de la acreditación observada en este estudio puede reflejar las expectativas más amplias de los estudiantes con respecto a la calidad educativa y la competencia profesional. Los estudiantes suelen considerar la acreditación como un indicador de la credibilidad institucional y del valor de los graduados en el mercado laboral, lo que podría explicar las actitudes favorables incluso cuando el conocimiento de los procedimientos es limitado. Sin embargo, la falta de conocimiento detallado que se destaca en la Tabla 3 sugiere que estas percepciones podrían basarse más en creencias generales que en una comprensión concreta. Esta desconexión subraya la importancia de integrar contenidos relacionados con la acreditación en los cursos formales y en las oportunidades de aprendizaje experiencial, a fin de garantizar que las percepciones de los estudiantes estén bien fundamentadas y alineadas con la práctica. Cabe señalar que la percepción no refleja necesariamente la competencia o el desempeño reales y, por lo tanto, los hallazgos deben interpretarse dentro de un marco perceptivo y educativo.

Los resultados del análisis de regresión brindan una comprensión más profunda de los factores determinantes de la percepción de la acreditación entre las estudiantes de partería. El conocimiento sobre la acreditación se reveló como el predictor más fuerte, lo que refuerza el papel central de la concientización en la formación de las percepciones de las estudiantes sobre los procesos de garantía de calidad. Este hallazgo concuerda con la idea de que comprender los procesos de calidad es fundamental para una participación significativa en la educación superior^(14)^. Los coeficientes de regresión indican que un mayor conocimiento de la acreditación por parte de los estudiantes y su disposición a participar están asociados con niveles más altos de percepción de la acreditación. Sin embargo, el modelo demostró un poder explicativo moderado (R^2^ = 0,173), lo que sugiere que otros factores también pueden contribuir a la percepción de la acreditación. Las investigaciones futuras podrían explorar variables adicionales, como la exposición clínica, el contexto institucional y las oportunidades de aprendizaje experiencial, para ofrecer una comprensión más integral de los determinantes de la percepción de la acreditación.

Este hallazgo indica que la percepción de la acreditación se basa en gran medida en el conocimiento y no en la experiencia, ya que ni la progresión académica ni la participación en actividades científicas influyeron significativamente en los niveles de percepción. Esto respalda la idea de que las competencias relacionadas con la acreditación no se desarrollan de manera pasiva con el tiempo, sino que requieren intervenciones educativas estructuradas.

Además, se identificó la disposición de los estudiantes a participar en los procesos de acreditación como un predictor significativo, lo que resalta la importancia de la participación estudiantil para fomentar actitudes positivas hacia el aseguramiento de la calidad. Este hallazgo concuerda con los marcos de calidad contemporáneos que posicionan a los estudiantes como partes interesadas activas en los procesos de calidad institucionales.

Este estudio cuenta con varios puntos fuertes, entre ellos un tamaño de muestra relativamente grande y el uso de una herramienta de medición validada con excelente consistencia interna. Sin embargo, deben reconocerse algunas limitaciones. El diseño transversal impide realizar inferencias causales, y el hecho de que el estudio se haya realizado en un solo centro puede limitar la generalizabilidad de los hallazgos. Además, los datos se basaron en medidas autoinformadas, lo que puede estar sujeto a sesgos de respuesta. Por otra parte, el modelo de regresión demostró un poder explicativo moderado, lo que indica que otras variables no medidas también pueden influir en la percepción de la acreditación.

Desde una perspectiva práctica, los hallazgos resaltan la importancia de integrar una formación estructurada sobre garantía de calidad y acreditación en los planes de estudio de pregrado en partería. Mejorar los conocimientos de los estudiantes e involucrarlos activamente en los procesos de acreditación puede contribuir al desarrollo de una cultura de calidad sostenible dentro de las instituciones educativas. Crear oportunidades para la participación de los estudiantes en actividades relacionadas con la calidad, como la evaluación de programas y los sistemas de retroalimentación, puede fortalecer aún más tanto las experiencias de aprendizaje como los esfuerzos institucionales de mejora de la calidad. Estos hallazgos indican que los conocimientos sobre acreditación pueden enseñarse de manera efectiva como parte de la educación de pregrado. Además, la participación de los estudiantes en los procesos de aseguramiento de la calidad puede estructurarse y apoyarse de manera sistemática, y la participación activa en dichos procesos puede contribuir al desarrollo profesional de los estudiantes.

Los conocimientos relacionados con la acreditación pueden integrarse en los planes de estudio a través de cursos o módulos estructurados, mientras que la participación de los estudiantes puede promoverse mediante su participación en comités de calidad y oportunidades de aprendizaje experiencial.

Este estudio contribuye a la literatura existente al destacar el papel del conocimiento y la participación de los estudiantes como determinantes clave de la percepción de la acreditación en la formación en partería, un área que ha recibido poca atención empírica. A diferencia de estudios anteriores que se centraron principalmente en los resultados a nivel institucional o de programa, este estudio ofrece una perspectiva centrada en los estudiantes sobre los procesos de acreditación. Los hallazgos ofrecen implicaciones prácticas para integrar contenidos relacionados con la acreditación en los planes de estudio y promover la participación activa de los estudiantes en los procesos de aseguramiento de la calidad, apoyando así el desarrollo de una cultura de calidad sostenible en la educación de las profesiones de la salud.

Estos hallazgos enfatizan la necesidad de estrategias educativas estructuradas que mejoren el conocimiento de los estudiantes sobre la acreditación y los involucren activamente en los procesos de aseguramiento de la calidad. Fortalecer la participación de los estudiantes en la acreditación no solo puede mejorar las experiencias educativas, sino también fomentar una cultura de calidad sostenible y mejores resultados de salud.

## CONCLUSIÓN

Este estudio demostró que las estudiantes de partería tienen un nivel moderado de percepción de la acreditación. Se identificó que el conocimiento sobre la acreditación y la disposición a participar en los procesos de acreditación son predictores significativos de la percepción de la acreditación, mientras que el año académico y la participación en actividades científicas no se asociaron con los niveles de percepción. Estos hallazgos indican que la percepción de la acreditación está determinada principalmente por el conocimiento y el compromiso, más que por la progresión académica. Fortalecer el conocimiento y el compromiso de los estudiantes podría contribuir al desarrollo de un entorno educativo más orientado a la calidad en la formación en partería.

## Data Availability

Los datos que respaldan los hallazgos de este estudio están disponibles a través del autor correspondiente previa solicitud razonable.
